# Methylation Regulation of *TLR3* on Immune Parameters in Lung Adenocarcinoma

**DOI:** 10.3389/fonc.2021.620200

**Published:** 2021-05-20

**Authors:** Ang Li, Hongjiao Wu, Qinqin Tian, Yi Zhang, Zhi Zhang, Xuemei Zhang

**Affiliations:** ^1^ School of Public Health, North China University of Science and Technology, Tangshan, China; ^2^ College of Life Science, North China University of Science and Technology, Tangshan, China; ^3^ School of Basic Medical Sciences, North China University of Science and Technology, Tangshan, China; ^4^ Affiliated Tangshan Gongren Hospital, North China University of Science and Technology, Tangshan, China

**Keywords:** TLR3, methylation, lung adenocarcinoma, immune infiltration, apoptosis

## Abstract

This study aims to analyze the methylation regulation of *TLR3* in lung adenocarcinoma (LUAD) and to explore the association of *TLR3* expression with immune microenvironment. *TLR3* has a decreased expression in LUAD tissues and low expression of *TLR3* is not only associated with poor prognosis in patients with LUAD, but also can be used as a diagnostic marker. Bisulfite sequencing PCR (BSP) results showed that the methylation level in the promoter of *TLR3* was negatively correlated with the level of *TLR3* mRNA in LUAD tissues. TIMER analysis showed that *TLR3* was negatively correlated with the tumor purity of LUAD and positively with immune cell infiltration to some extent. ESTIMATE analysis also suggested that *TLR3* expression and its methylation had significant correlation with immune score. The lower immune scores were associated with the late stage of LUAD and poor prognosis. The high expression of *TLR3* might inhibit the development of LUAD by activating apoptosis pathway. The proteins interacted with *TLR3* were mainly involved in the apoptosis pathway and positively correlated with the key genes (*MYD88*, *Caspase 8*, *BIRC3*, *PIK3R1*) in this pathway. Therefore, *TLR3* as a key biomarker for prognosis and diagnosis in LUAD, might be considered as a potential epigenetic and immunotherapeutic target.

## Introduction

Lung adenocarcinoma (LUAD) is the major histological subtype of non-small cell lung cancer which accounts for about 80–85% of all lung cancer (1). Due to the lack of clinical symptoms and effective screening techniques, most patients are diagnosed at late-stage and have a poor prognosis. Therefore, the effective LUAD biomarkers are still needed to be developed.

Toll-like receptors (TLRs) are one of important pattern recognition receptors which have been demonstrated to be associated with the tumor progress. Several of TLR agonists have been investigated in clinical studies for their potential function in antitumor immunity (2–4). Toll-like receptor 3 (*TLR3*), as one of important TLR members, is participated in the development of many tumors by recognizing and binding to its corresponding ligands (5–9). *TLR3* has been reported to express on normal epithelial cells, such as lung tissue. The abnormal expression of *TLR3* has been reported on various of cancer tissues (10, 11). *TLR3* has also been recognized as a favorable prognosis biomarker of lung cancer by activating apoptosis or promoting autophagy (12, 13). *TLR3* agonist enhanced T cell infiltration in lung tissue, which is essential for tumor immunity within the tumor microenvironment (14–16). A clinical trial showed that Poly-ICLC (Hiltinol), an advanced form of *TLR3* agonist, could kill cancer cells and inhibit the metastasis of non-small cell lung cancer by activating several cancer suppressors and rebuilding an immunosuppressive tumor microenvironment (17).

Tumor microenvironments (TMEs) are largely populated with stromal and immune cells, which play the crucial role in the initiation and progression of lung cancer (18). Studies revealed that the proportion and character of T lymphocytes within TMEs had important effects on the progression and prognosis of lung cancer (16, 19, 20). Studies also revealed that the innate immune cells, such as macrophages and dendritic cells, when presenting in TMEs, contributed to the lung cancer initiation and played a key role in cancer immunotherapy (21–23). The crosstalk between cancer cells and the proximal immune cells ultimately is favorable to foster tumor growth and metastasis (24).

DNA methylation, one of Epigenetic mechanisms, plays a crucial role in controlling gene expression. The aberrant alteration of methylation has been demonstrated to be associated with the development of lung cancer (25, 26). The alterations of DNA methylation promote tumorigenesis by silencing tumor suppressor genes (27). Several candidate methylation biomarkers have been identified to serve as lung cancer detection and prognostic evaluation (28–30).

The anti-cancer activity induced by TLRs is partly due to the functional regulation of immune cells infiltrating in TME (31, 32). In order to investigate the interaction between tumor cells and tumors microenvironment, we used estimation of stromal and immune cells in malignant tumor tissues using expression data (ESTIMATE) algorithm to estimate the stromal and immune cells scores in LUAD and further evaluated the association of *TLR3* expression with immune microenvironment. We also evaluated the effects of *TLR3* on LUAD development and prognosis and to further shed light on the methylation regulatory of *TLR3*.

## Materials and Methods

### Patients and Tissue Samples

Tumor and its normal adjacent lung specimen from 20 patients with primary lung adenocarcinoma were collected from Affiliated Tangshan Gongren Hospital of North China University of Science and Technology. The detailed clinicopathological characteristics of these cases are summarized in **Table 1**. This study was approved by the Institutional Review Board of North China University of Science and Technology and informed consent was signed by all participants.

**Table 1:** Clinicopathological characteristics of the patients.

Variables	Number of cases (%)
Number of patients	20 (100)
Age (years)	
≤60	6 (30)
>60	14 (70)
Gender	
Male	11 (55)
Female	9 (45)
Smoking	
Yes	8 (40)
No	12 (60)
Drinking	
Yes	7 (35)
No	13 (65)
Tumor stage	
Stage I + II	13 (65)
Stage III + IV	7 (35)
Primary tumor	
T1–2	17 (85)
T3–4	3 (15)
Regional lymph nodes	
N0	16 (80)
N1-3	4 (20)

### Prognosis Analysis

To measure the separability by *TLR3* mRNA, receiver operating characteristics (ROC) curve was drawn and the area under curve (AUC) was calculated by “pROC” package. Based on the best separation cut-off value, overall survival (OS) and progression-free survival (PFS) was analyzed by KM Plotter Online Tool.

### Cell Culture and Treatment

The lung cancer cell lines (A549 and NCI-H460) and human normal bronchial epithelial cell BEAS-2B were all bought from American Type Culture Collection (ATCC). A549 and NCI-H460 cells were cultured in RPMI-1640 medium and BEAS-2B cells was cultured in DMEM medium. All mediums were mixed with 10% fetal bovine serum (FBS; Life Technologies, NY, USA) and antibiotics (100 U/ml penicillin and 100 μg/ml streptomycin) in a humidified incubator with 5% CO_2_ at 37°C. For *TLR3* agonist treatment, 10 and 50 µg/ml Poly (I:C) (ThermoFisher, USA) were used to treat A549 cells for 48 h.

### Bisulfite Sequencing and DNA Demethylation Treatment

Genomic DNA was extracted from lung cancer cells and subjected to sodium bisulfite modification using EpiTect Bisulfite Kits (TIANGEN, Beijing, China) following the manufacturer’s protocols. Sodium bisulfite-treated DNA was amplified using Bisulfite sequencing PCR (BSP) primers (5’-AGT ATT TTG GGA GGT TAA GGT GG-3’ and 5’-AAT AAC AAA CCA AAT ATA ATT AAC AAA T-3’). PCR products were subcloned into pGM-T vector according to the manufacturer’s instruction. Ten colonies from each sample were then picked up for sequencing. To test the effect of methylation on the expression of *TLR3*, the methylation inhibitor 5-Aza-2-deoxycytidine (Sigma, CA, USA) was used to treat lung cancer cells (A549 and NCI-H460) in different concentration (0, 5, 10 μmol/L) for 72 h.

### RNA Extraction and qRT-PCR

Total RNA was extracted from 20 pairs of LUAD and matched para-cancer tissues by using Trizol reagent (Invitrogen, CA, USA) and were then reversely transcribed into cDNA with RevertAid First Strand cDNA Synthesis Kit (Thermo Fisher Scientific, NY, USA). *TLR3* mRNA was detected using Power SYBR Green PCR Master Mix (Thermo Fisher Scientific, NY, USA) in ABIPRISM^®^ 7900HT Fast Real-Time PCR System (Applied Biosystems, Foster City, USA). The amplification procedure was: 50°C for 2 min, 95°C for 2 min, followed by 40 cycles of 95°C for 15 s and 60°C for 2 min. *GAPDH* was used as the reference gene. The relative mRNA expression was analyzed using the 2^−△△Ct^ method. The experiment was repeated three times. The qRT-PCR primers of *TLR3* are as follows: 5’-CAA ACA CAA GCA TTC GGA ATC TG-3’ and 5’-AAG GAA TCG TTA CCA ACC ACA TT-3’.

### Stromal/Immune Scores and Tumor-Infiltrating Immune Cells Infiltration

ESTIMATE is a newly developed algorithm that takes advantage of the unique properties of the transcriptional profiles of cancer tissues to infer tumor cellularity as well as the different infiltrating normal cells (33). We used this algorithm to impute stromal and immune scores and then to predict the level of infiltrating stromal and immune cells based on the gene expression profile. Furthermore, the abundance of infiltrating immune cells was evaluated by Tumor Immune Estimation Resource (TIMER) algorithm. For survival analysis, LUAD patients were classified by stromal/immune scores after getting the optimal cutoff value by X-tile Software.

### Western Blot Analysis

Lung cancer cells were lysed in a lysis buffer and protein samples were run on 8% SDS polyacrylamide gel and then transferred to Nitrocellulose (NC) membrane. After incubated with the specified primary antibody, and then corresponding horseradish peroxidase (HRP) conjugated secondary antibody, specific protein was developed by enhanced chemiluminescence (ECL) luminescence reagents (Amersham, UK). *β-actin* was applied as reference control. All antibodies (anti-PI3 kinase p85 alpha, anti-caspase 8, anti-MyD88 and anti-BIRC3 antibodies) were purchased from Abcam (Eugene, USA).

### Gene Enrichment and GSVA Analysis

We used the “ggplot2” R package and the DAVID 6.8 database (https://david.ncifcrf.gov/) to conduct Gene Ontology (GO) and Kyoto Encyclopedia of Genes and Genomes (KEGG) analysis. Using TCGA-LUAD RNA-seq data, we performed the Gene Set Variation Analysis (GSVA) using GSVA R package to determine the pathways which most related to *TLR3*. The gene set “2.cp.kegg.v6.2.symbols.gmt” from the Molecular Signature Database was selected as the reference.

### Statistical Analyses

All analyses were performed with R version 3.6.1 and its appropriate packages. Statistical significance of Student’s t-test was used for two-group comparisons. Survival analysis was estimated by the Kaplan–Meier method and the log-rank test. *P <*0.05 was considered as significant.

## Results

### The Expression and Clinical Significance of *TLR3* in Human LUAD

**Figure 1:**
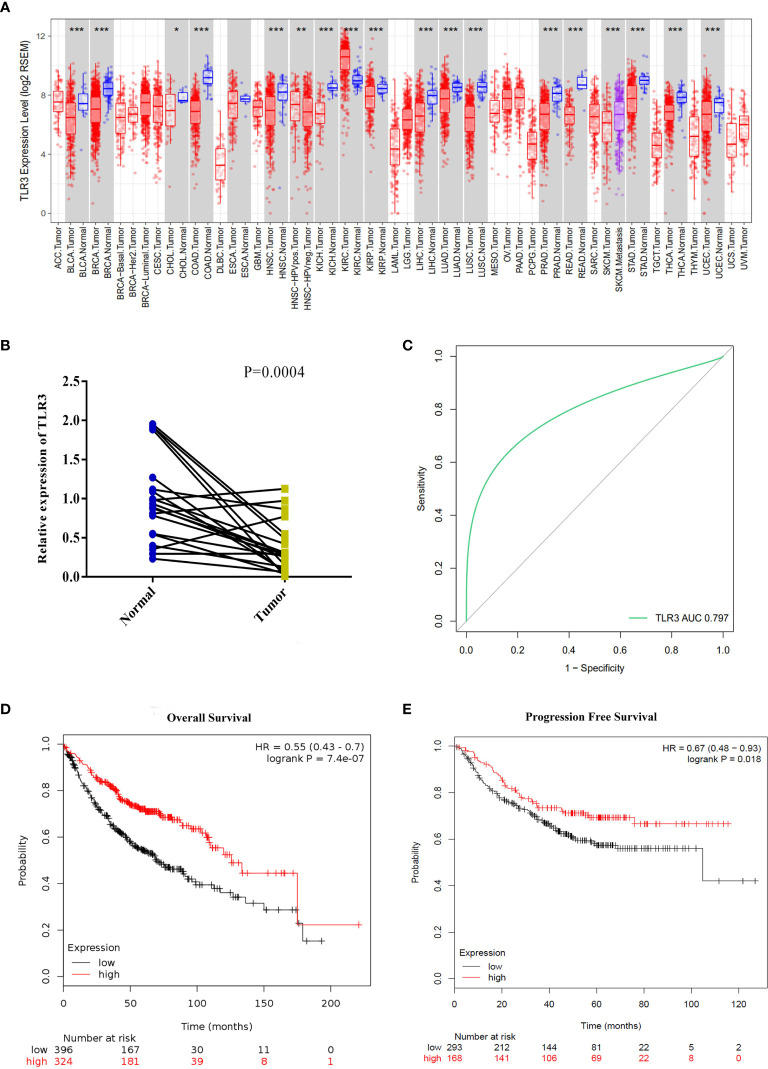
*TLR3* expression levels in lung adenocarcinoma and its relationship with prognosis. **(A)** *TLR3* expression levels in different tumor types from TCGA database were determined by TIMER (*P < 0.05, **P < 0.01, ***P < 0.001). **(B)** Quantification of *TLR3* expression levels in lung adenocarcinoma and paired adjacent normal tissues by qRT-PCR. P-values were obtained by Student’s t-test. **(C)** ROC curve from qRT-PCR shows *TLR3* is a marker to distinguish lung adenocarcinoma tissues from normal tissues. **(D, E)** Overall survival and disease-free survival analysis of lung adenocarcinoma patients based on *TLR3* expression. Data was analyzed using Kaplan–Meier Plotter.

To determine the differential expression of *TLR3*, we analyzed the data from the cancer genome atlas (TCGA) by TIMER and found that *TLR3* was significantly decreased in LUAD tissues than in adjacent lung tissues (**Figure 1A**). To verify the result from TCGA, we measured the mRNA level of *TLR3* in 20 paired LUAD tissues and adjacent normal tissues by qRT-PCR which turned out to be consistent with the finding of database (**Figure 1B**).

To measure the separability by the expression of *TLR3*, ROC curve was plotted (**Figure 1C**). The area under the curve for *TLR3* expression was 0.806, which means a good measure of separability by *TLR3*. Using the Kaplan–Meier plotter data, we found that the expression of *TLR3* was positively correlated with the overall survival (HR = 0.55, 95%CI = 0.43–0.7, *P* = 7.4e−07) and progression-free survival (HR = 0.67, 95%CI = 0.48–0.93, *P* = 0.018) in LUAD cancer patients (**Figures 1D, E** **)**.

### *TLR3* Is Regulated by DNA Methylation

**Figure 2:**
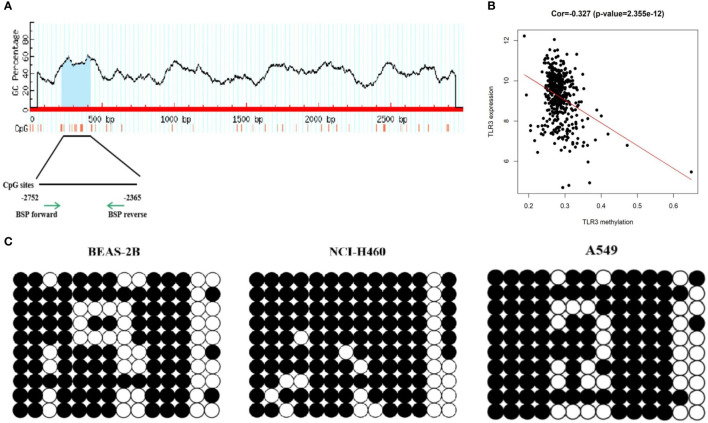
DNA methylation detection of *TLR3* promoter. **(A)** MethPrimer database predicts CpG islands in the *TLR3* promoter region. **(B)** Pearson correlation analysis of *TLR3* expression and methylation degree. **(C)** BSP analysis of methylation status in the *TLR3* promoter region.

Using the MethPrimer program, we found that there was a CpG island with fourteen CpG sites in the proximal promoter region (3,000 bp upstream the TSS site) of *TLR3* (**Figure 2A**). Upon analyzing TCGA methylation data of LUAD, we found that *TLR3* methylation was negatively correlated with the level of *TLR3* mRNA (**Figure 2B**). The sequencing of bisulfite converted DNA presented a higher methylation level in A549 and NCI-H460 cancer cells than that in BEAS-2B cells (**Figure 2C**).

**Figure 3:**
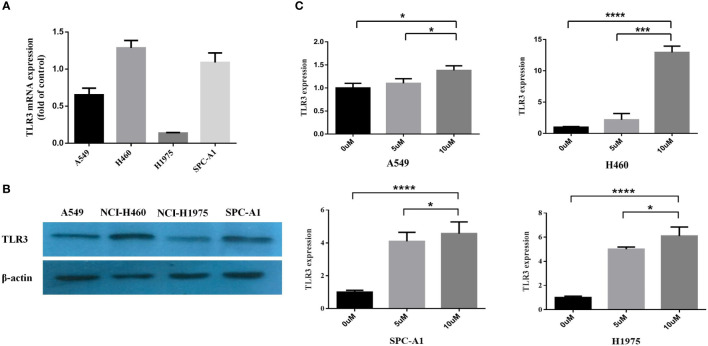
*TLR3* expression is regulated by DNA methylation of its promoter. **(A, B)** The expression levels of *TLR3* mRNA and protein were measured by qRT-PCR and western blot analysis in A549, NCI-H460, NCI-H1975 and SPC-A1 cells, respectively. **(C)** The expression of *TLR3* restored in A549, NCI-H460, NCI-H1975 and SPC-A1 cell lines after treatment with 5-aza-2′-deoxycytidine for 72 h. Data are from at least three independent experiments. *P < 0.05, ***P < 0.001, ****P < 0.0001.

After detecting the mRNA and protein level of *TLR3* in lung cancer cells (A549, NCI-H460, NCI-H1975 and SPC-A1) (**Figures 3A, B** **)**, we investigated the effect of methylation on the expression of *TLR3*. We treated lung cancer cells with methyltransferase inhibitor 5-aza-dc and found that *TLR3* mRNA was significantly increased in dose-dependent manner (**Figure 3C**).

### Estimation of Infiltrating Cells and Tumor Purity and Their Impact on LUAD Progress and Prognosis

**Figure 4:**
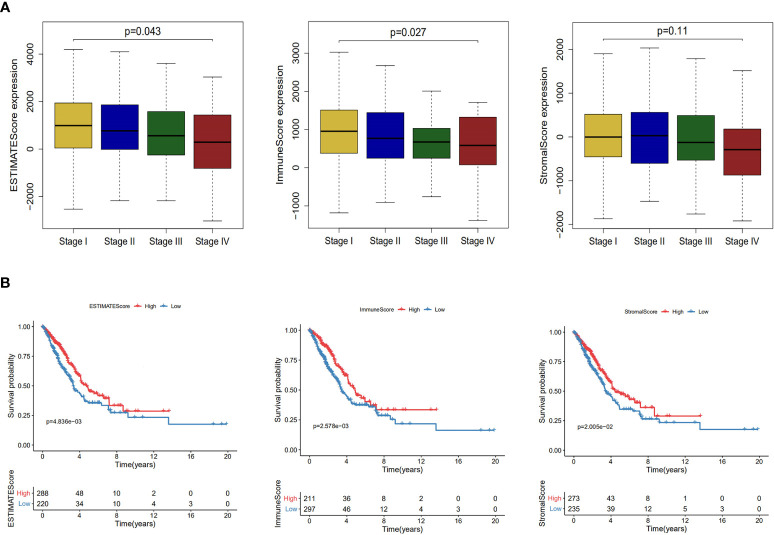
Association of stromal and immune scores with lung adenocarcinoma pathology and prognosis. **(A)** Distributions and comparisons of ESTIMATE score, immune score and stromal score among different tumor stages. **(B)** Kaplan–Meier plot of overall survival for patients with ESTIMATE score, immune score and stromal score.

In tumor microenvironment, infiltrating stromal and immune cells play an important role in the development, progression and prognosis of lung cancer (15, 34). ESTIMATE algorithm was performed for each sample to predict the abundance of infiltrating stromal and immune cells using immune and stromal scores and to infer tumor purity using ESTIMATE score (33). Samples with higher stromal or immune scores were represented for larger amount of the immune or stromal components in tumor microenvironment. After estimating the stromal, immune and ESTIMATE scores, we evaluated the impact of these scores on the progression and prognosis of LUAD. As shown in **Figure 4A**, immune scores and ESTIMATE scores roughly decreased with increasing tumor stage (*P* = 0.027; *P* = 0.043). After separated into two groups by immune/stromal/ESTIMAT score, patients with higher these scores had significantly longer overall survival time (*P* = 4.83e−03, 5.76e−02 and 3.74e−02) (**Figure 4B**).

### *TLR3* Expression Is Correlated With Immune Cells Infiltrating and Tumor Purity in LUAD

**Figure 5:**
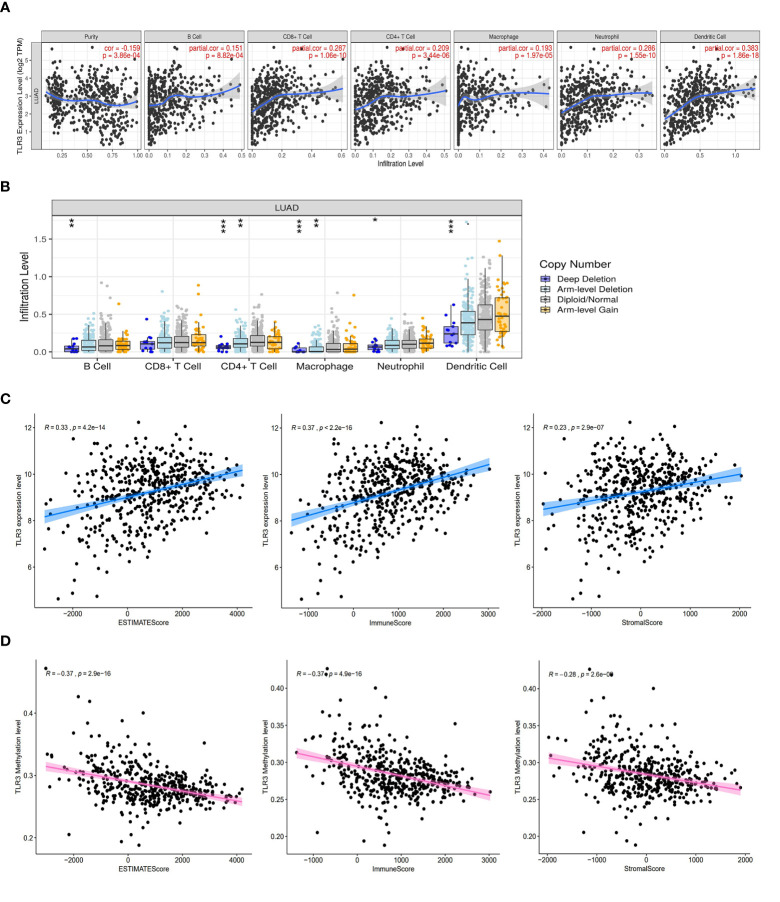
*TLR3* expression is correlated with immune cells infiltrating and tumor purity in LUAD. **(A)** Correlation analysis of *TLR3* expression and immune cells. **(B)** Correlation analysis of *TLR3* copy number variation and immune cells. **(C)** *TLR3* expression was positively correlated with ESTIMATE score, immune score and stromal score in lung adenocarcinoma. **(D)** *TLR3* methylation was negative correlated with ESTIMATE score, immune score and stromal score in lung adenocarcinoma. *P < 0.05, **P < 0.01,***P < 0.001.

Due to the important role of toll-like receptors in innate immunity, we analyzed the relationship between *TLR3* expression and immune cell infiltration by TIMER (**Figure 5A**) and found that *TLR3* expression was related to infiltrating levels of B cells (r = 0.151, *P* = 8.82e−04), CD8^+^ T cells (r = 0.287, *P* = 1.06e−10), CD4^+^ T cells (r = 0.209, *P* = 3.44e−06), Macrophages (r = 0.193, *P* = 1.97e−05), Neutrophils (r = 0.286, *P* = 1.55e−10), and Dendritic cells (r = 0.383, *P* = 1.86e−18) in LUAD. We also found that *TLR3* copy number variation (CNV) had effect on the infiltration level of several immune cells (B cell, CD4^+^ T cells, Macrophages, Neutrophils and Dendritic cells) (**Figure 5B**).

To further evaluate the impact of *TLR3* on the level of immune cells infiltrating and tumor purity, we analyzed the correlation between *TLR3* and the immune/stromal/ESTIMAT score. Our results showed that stromal score, immune score and ESTIMATE score in LUAD were positively associated with *TLR3* expression, but negatively associated with *TLR3* methylation (**Figures 5C, D** **)**.

### The Potential Mechanism of *TLR3* in LUAD Development

**Figure 6:**
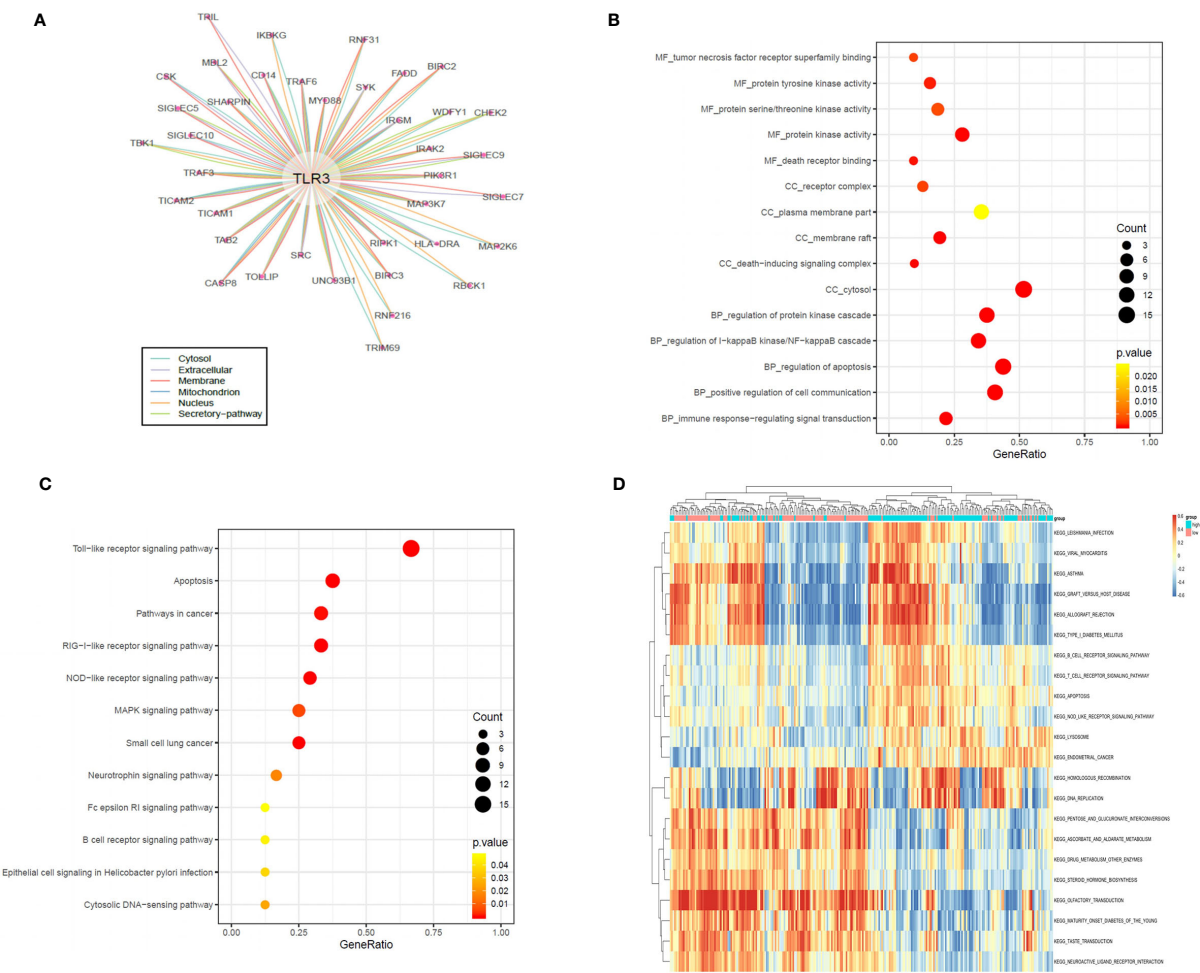
*TLR3* regulate LUAD development by apoptotic pathways **(A)**. Protein–protein interaction (PPI) analysis of the network linking *TLR3* by the ComPPI database. **(B, C)**. *TLR3* interacting proteins performing GO function enrichment and KEGG pathway analysis. **(D)** GSVA-derived clustering heatmaps of differentially expressed pathways for *TLR3*. Only signaling pathways with log(foldchange) > 0.2 are shown.

**Figure 7:**
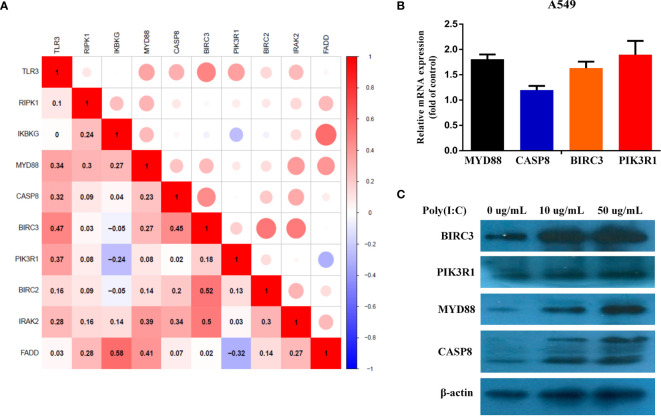
Evaluate the correlation between different *TLR3* expression and apoptosis genes. **(A)**. Pearson Correlation analysis of *TLR3* and apoptosis genes. **(B, C)**. The expression levels of *MYD88*, *CASP8*, *BIRC3* and *PIK3R1* mRNA and protein were measured by qRT-PCR and western blot analysis after 24 h Poly(I:C) treatment in the A549 cells, respectively.

To discover the potential mechanism of *TLR3* in the development of LUAD, we used the compartmentalized protein-protein interaction database (ComPPI) to construct the protein–protein interaction network of *TLR3*. As a result, a total of 38 central interactive proteins were filtered out with an average Interaction Score of 0.749 (**Figure 6A**). Based on these interactive proteins, we performed GO and KEGG enrichment analysis. The enrichment analysis showed that these proteins were involved in apoptosis pathway (**Figures 6B, C** **)** which contained nine genes, including receptor-interacting protein kinase 1(*RIPK1*), inhibitor of nuclear factor kappa B kinase regulatory subunit gamma (*IKBKG*), myeloiddifferentiationfactor88 (*MYD88*), Caspase 8, baculoviral iap repeat containing 3 (*BIRC3*), phosphoinositide-3-kinase regulatory subunit 1 (*PIK3R1*), baculoviral iap repeat containing 2 (*BIRC2*), interleukin 1 receptor associated kinase 2 (*IRAK2*) and fas associated *via* death domain (*FADD*). LUAD samples were divided into two groups by the median of *TLR3* mRNA level. GSVA confirmed that most samples were enriched in pathways closely correlated with tumorigenesis, including apoptosis (**Figure 6D**). We then evaluated the relationship between *TRL3* and these apoptosis-related genes in RNA level using TCGA dataset. The results turned out a significant correlations between*TLR3* and the expression of *MYD88*, *Caspase 8*, *BIRC3* and *PIK3R1* (r >0.3; *P <*0.001) (**Figure 7A**). To further verify the above results, we used *TLR3* agonist Poly(I:C) to treat lung cancer cells (A549) and found that the expressions of *MYD88*, *Caspase 8*, *BIRC3* and *PIK3R1* were in line with the expression of *TLR3* (**Figures 7B, C** **)**.

## Discussion

Toll-like receptors are one of the most well-known pattern recognition receptor family members. *TLR3* is the only one member of TLRs which signals through *TRIF* instead of *MyD88*. In recent years, *TLR3* has been reported to be aberrantly expressed in several cancer tissues and is involved in cancer therapy and prognosis of certain cancers. For example, *TLR3* resisted the DNA damage induced by cisplatin, which leads to the resistance of head and neck cancer cells to cisplatin (5). *TLR3* expression in neuroblastoma was associated with the improving prognosis (35). The suppression of *TLR3* was related to tumor proliferation, angiogenesis, and the inhibition of apoptosis in liver cancer (6, 10). However, several reports also demonstrated that *TLR3* protein level on tumor cells was associated with improving overall survival in a stage-I cohort study of non-small cell lung cancer (36). Similarly, in our current study, we presented that *TLR3* was not only downregulated, but also served as an independent prognostic marker for LUAD patients. The low expression of *TLR3* is considered to be related to a poor outcome of LUAD. Go and KEGG enrichment analyses presented that *TLR3* was correlated with several key proteins in the apoptotic pathway. GSVA analysis showed that highly expressed *TLR3* could also activate the apoptotic pathway.

As one of important epigenetic mechanisms, DNA methylation has received more and more attention in recent decades. Hypermethylation in gene promoter regions adjacent to transcription start sites (TSSs) often leads to gene silencing, and then result in the alterations of gene function in several cancer types (37, 38). Some studies have shown that DNA methylation was widely used for early diagnosis and treatment in breast cancer, gastric cancer, and liver cancer (39). In this study, we systematically evaluated the relationship between DNA methylation and the expression of *TLR3* in LUAD and found that the hypermethylation of the *TLR3* inhibited the activity of the gene. The expression of *TLR3* increased when LUAD cells were treated with 5-Aza-dc. These results provided new insights into the role of DNA methylation in the regulation of LUAD.

Tumor progression is also affected by its extrinsic tumor microenvironment. Studies have shown that TIME played key roles in regulating the process of carcinogenesis, tumor invasion, and metastasis (40). The immune suppressive microenvironment induced by tumor infiltrating regulatory T lymphocytes prevents effective anti-tumor immunity and has become the key factor of immunotherapy against cancer (41–43). Studies also showed that LUAD had higher levels of immune infiltration than other histopathological types, therefore LUAD patients were more sensitive to immunotherapy (44, 45). TLRs are expressed not only on human tumor cells, but also on immune cells (46–48). As one of the important TLR family members, *TLR3* have been confirmed to promote the activation of CD8^+^ T lymphocytes and *IFN* production in dendritic cells by binding to specific ligands (49, 50). Noteworthy, our results indicated a substantial explicit connection between *TLR3* expression and infiltration levels of immune cells in LUAD.

TME is a complicated system, in which immune and stromal cells are two major non-tumor components which are associated with tumor growth and prognosis (51, 52). ESTIMATE algorithm has been used to predict the purity of tumors and the levels of infiltrating matrix and immune cells in tumor tissues (33, 53–57). A recent meta-analysis with 29 studies indicated that tumor-infiltrating immune cells had a superior prognostic impact on lung cancer (58). In current study, we demonstrated that LUAD patients with late clinical pathological stages or poor prognosis had low-stromal and immune scores. Our data also presented a positive correlation between stromal/immune scores and *TLR3* expression. These results suggested that increased *TLR3* level might inhibit cancer cell proliferation by activating immune cells infiltrating.

Taken together, our findings serve for a better understanding of the role of *TLR3* in the development of lung cancer. Furthermore, if confirmed in further in-deep study, *TLR3* could be considered as a promising lung cancer biomarker for tumor aggressiveness and prognosis.

## Data Availability Statement

The original contributions presented in the study are included in the article/supplementary materials. Further inquiries can be directed to the corresponding author.

## Ethics Statement

The studies involving human participants were reviewed and approved by Institutional Review Board of North China University of Science and Technology. The patients/participants provided their written informed consent to participate in this study.

## Author Contributions

AL drafted the manuscript. AL and YZ performed the bioinformatic analysis. AL and QT conducted methylation analysis. ZZ and HW performed statistical analyses. ZZ collected tissue samples. XZ designed and supervised the research. All authors contributed to the article and approved the submitted version.

## Funding

This study was supported by Key Project of Natural Science Foundation of Hebei province of China (grant number H2017209233).

## Conflict of Interest

The authors declare that the research was conducted in the absence of any commercial or financial relationships that could be construed as a potential conflict of interest.
